# Transcutaneous fluorescence spectroscopy: development and characterization of a compact, portable, and fiber-optic sensor

**DOI:** 10.1117/1.JBO.29.2.027003

**Published:** 2024-02-28

**Authors:** Elena Monfort Sanchez, James Avery, Jonathan Gan, Jingjing Qian, Nilanjan Mandal, Arjun Agarwal, Mulima Mwiinga, Rose Banda, Ara Darzi, Paul Kelly, Alex J. Thompson

**Affiliations:** a Institute of Global Health Innovation, Imperial College London, The Hamlyn Centre, London, United Kingdom; b St. Mary’s Hospital Campus, Imperial College London, Department of Surgery and Cancer, London, United Kingdom; c University of Zambia School of Medicine, Tropical Gastroenterology and Nutrition Group, Lusaka, Zambia; d Queen Mary University of London, Blizard Institute, London, United Kingdom

**Keywords:** optical sensing, transcutaneous fluorescence spectroscopy, noninvasive, gut permeability

## Abstract

**Significance:**

The integrity of the intestinal barrier is gaining recognition as a significant contributor to various pathophysiological conditions, including inflammatory bowel disease, celiac disease, environmental enteric dysfunction (EED), and malnutrition. EED, for example, manifests as complex structural and functional changes in the small intestine leading to increased intestinal permeability, inflammation, and reduced absorption of nutrients. Despite the importance of gut function, current techniques to assess intestinal permeability (such as endoscopic biopsies or dual sugar assays) are either highly invasive, unreliable, and/or difficult to perform in certain patient populations (e.g., infants).

**Aim:**

We present a portable, optical sensor based on transcutaneous fluorescence spectroscopy to assess gut function (in particular, intestinal permeability) in a fast and noninvasive manner.

**Approach:**

Participants receive an oral dose of a fluorescent contrast agent, and a wearable fiber-optic probe detects the permeation of the contrast agent from the gut into the blood stream by measuring the fluorescence intensity noninvasively at the fingertip. We characterized the performance of our compact optical sensor by comparing it against an existing benchtop spectroscopic system. In addition, we report results from a human study in healthy volunteers investigating the impact of skin tone and contrast agent dose on transcutaneous fluorescence signals.

**Results:**

The first study with eight healthy participants showed good correlation between our compact sensor and the existing benchtop spectroscopic system [correlation coefficient (r)>0.919, p<0.001]. Further experiments in 14 healthy participants revealed an approximately linear relationship between the ingested contrast agent dose and the collected signal intensity. Finally, a parallel study on the impact of different skin tones showed no significant differences in signal levels between participants with different skin tones (p>0.05).

**Conclusions:**

In this paper, we demonstrate the potential of our compact transcutaneous fluorescence sensor for noninvasive monitoring of intestinal health.

## 1 Introduction

Disruption of the intestinal barrier manifests in numerous gastrointestinal (GI) conditions, including inflammatory bowel disease (IBD), celiac disease, and environmental enteric dysfunction (EED). This can lead to an increase in gut permeability and potentially the translocation of bacteria and other GI pathogens into the systemic circulation causing inflammatory responses.^1^^,^^2^ EED, for example, is a poorly understood condition characterized by multifarious changes in the structure and function of the small intestine, including inflammation, reduced villous height, and increased intestinal permeability (or “leakiness”).^1^^,^^2^ Interestingly, similar changes in intestinal function (including increased permeability) have also been reported in a wide range of clinical conditions both within and outside of the GI tract (including IBD, celiac disease, and liver disease).^2^^–^^5^

Despite the biological and clinical importance of intestinal permeability, current clinical tests suffer from a series of limitations. The most common approaches include endoscopic biopsy and histopathology, as well as chemical tests such as the lactulose:mannitol (L:M) and lactulose:Rhamnose (L:R) assays. However, the clinical uptake of these tests is limited as they are either too cumbersome, too invasive, nonstandardized, or difficult to perform in certain patient groups (e.g., L:M and L:R assays are challenging in infants^5^ and in patients with reduced urinary output^6^). In addition, laboratory-based examination of the collected samples is required in all cases, which leads to a delay between administering the tests and reporting results.

These disadvantages—in conjunction with the lack of understanding of the role of the gut in the above conditions and diseases—demonstrate the need for noninvasive and reliable diagnostic tools to provide improved assessment and monitoring of intestinal permeability (and other aspects of gut function).^7^^–^^9^

Recent clinical and preclinical studies have demonstrated that transcutaneous “through-the-skin” fluorescence spectroscopy of orally ingested fluorescent contrast agents has the potential for noninvasive monitoring of gut permeability and other GI functions.^10^^–^^15^ This approach entails subjects receiving an oral dose of a fluorescent contrast agent and a fluorescence sensing probe being placed on the skin to detect the permeation of that agent from the gut into the blood stream.^10^^–^^15^ Fluorescein (a fluorescent contrast agent that is approved for use in many clinical settings^16^) is expected to be suitable for transcutaneous assessment of gut function as it has a molecular weight that is comparable to lactulose (which is widely used to measure gut barrier function, e.g., in the L:M and L:R tests). Transcutaneous spectroscopy has the potential to produce clinical results within hours rather than days (as data can be analyzed immediately without requiring laboratory analysis) and does not require collection of urine, blood, or stool samples (unlike other methods such as L:M and L:R).^10^^–^^15^

However, to date, transcutaneous fluorescence spectroscopy to measure gut permeability has only been deployed clinically using laser-based spectrometers and microscopes (hereafter referred to as benchtop systems), which are both large and expensive.^10^^–^^13^ The use of these benchtop systems can therefore introduce additional challenges in certain patient groups (e.g., children/infants) and in certain environments (such as low-income countries), thereby precluding widespread deployment.

To address these issues, in this article, we present a compact fluorescence sensor for noninvasive assessment of gut permeability via transcutaneous spectroscopy. We compare the performance of the compact optical sensor against a clinically deployed benchtop system that was previously presented in the literature.^10^^,^^11^^,^^13^ In addition, we report preliminary data from a clinical study on healthy participants investigating the impact of skin tone and contrast agent dose on transcutaneous fluorescence signals, thereby demonstrating the potential of the compact sensor for noninvasive monitoring of gut function.

## 2 Material and Methods

### 2.1 Compact Fiber-Optic Fluorescence Sensor—Optical Setup

As discussed above, transcutaneous fluorescence spectroscopy entails the oral ingestion of a fluorescent contrast agent (the clinically approved dye fluorescein was used in all experiments presented here) and the noninvasive measurement (using a sensor placed in contact with the skin) of the permeation of that contrast agent from the gut into the blood stream. The resulting data can then be analyzed to facilitate measurements of gut permeability and other clinically relevant GI functions.

To allow for more widespread deployment of transcutaneous spectroscopy to assess gut permeability, we developed a compact fluorescence spectroscopy sensor to evaluate fluorescence signals at the fingertip. Although compact and even wearable transcutaneous fluorescence sensors exist (e.g., Ref. 17), we developed a fiber-based system here to facilitate the closest possible spacing between the light source and detector. This provides the highest possible sensitivity, which is crucial for the assessment of gut function in which the concentration of fluorescent dye in the blood stream is typically low compared with applications in other organs (e.g., in the kidney or liver where dyes are injected into the blood stream).

**Fig. 1:**
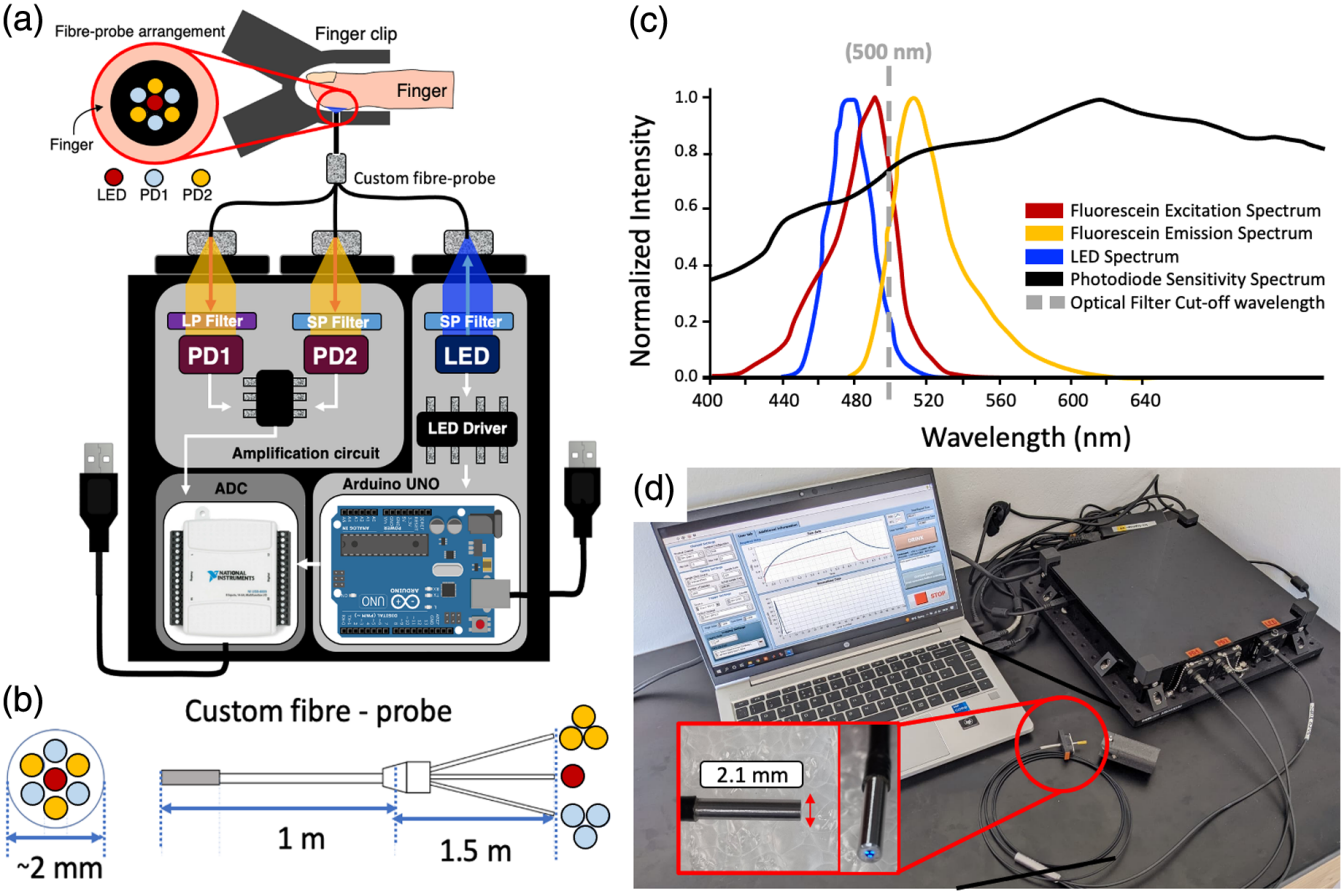
Compact fluorescence sensor for transcutaneous assessment of gut function. (a) Schematic diagram of the compact fluorescence sensor. (b) Custom fiber probe diagram showing distal fiber arrangement (left) and full bundle (right). (c) Excitation/emission/sensitivity spectra of optical components (including fluorescent contrast agent) used in the compact fluorescence sensor; data extracted from component datasheets. (d) Photograph of the compact sensor deployed in a clinical environment. The laptop shows the user interface developed for collection and preprocessing of data. The interface is shown in more detail in Fig. S1 in the Supplementary Material. A 3D printed finger clip is shown next to the fiber tip. A close-up of the distal tip of the optical fiber probe is shown in the insert (red box).

A schematic diagram of the optical setup for our compact sensor is shown in Fig. 1(a). The optical part of the sensor comprises a 465 nm light-emitting diode (LED; LST1-01H06-BLU1-01, New Energy) for excitation and two photodiodes (2 × SFH 2716 A01, ams OSRAM, New Energy; peak sensitivity at 620 nm, ∼80% relative sensitivity at 520 nm) to detect the fluorescence signal (photodiode 1) and the backscattered excitation signal (photodiode 2). Hereafter, the photodiodes will be referred to as the fluorescence photodiode (photodiode 1) and the backscatter photodiode (photodiode 2). The signal collected by the backscatter photodiode is used to correct for variations due to, for example, changes in probe orientation, excitation power fluctuations, and differences in skin color and/or skin thickness.

The light source (LED) and the detectors (photodiodes) are coupled to a custom trifurcated fiber-optic probe (2 m total length; FiberTech Optica, Inc.) to allow for interrogation of fluorescence signals at the skin. The custom probe contains seven multimode silica optical fibers (200  μm core diameter); the arrangement of fibers in the probe is depicted in Fig. 1(b). The common distal end of the custom probe has a single excitation fiber located in the center of the probe surrounded by a ring of six fibers for light collection. The proximal end has three independent channels that are connected to the LED and the two photodiodes, respectively. The single optical fiber routes the light from the LED to the measurement site (typically the fingertip). Of the six collection fibers, three are connected to the fluorescence photodiode and three are connected to the backscatter photodiode. The collection fibers are interleaved at the distal tip to ensure that the interrogation volume is the same for both photodiodes. The center–center spacing of fibers at the distal tip is 250  μm, which is the same as that in the benchtop system^10^^,^^11^^,^^13^ used for validation (see Sec. 2.4).

Flexible reflective optical filters (Everix Ultra-Thin OD 2 longpass/shortpass filters, cut-off/on wavelength: 500 nm, Edmund Optics) were positioned in front of the LED and the photodiodes. Shortpass filters were used to clean up the LED emission profile and to stop any fluorescence signal from reaching the backscatter photodiode. Similarly, longpass filters were used to stop the backscattered excitation light from reaching the fluorescence photodiode. The spectral properties of the optical components are presented in Fig. 1(c).

Two filters were used in front of each component (i.e., LED and photodiodes) to ensure sufficient light rejection. Figure S2 in the Supplementary Material shows the suppression of excitation light achieved using optical configurations with one and two short- and longpass filters, respectively (graphs in Fig. S2 in the Supplementary Material show spectra observed when LED excitation light was directed through the optical filters onto a spectral detector; FLAME, Ocean Insight). The use of one shortpass and one longpass filter resulted in clear detection of the residual signal from the LED, whereas the two-filter configuration provided near-total suppression of the excitation light (Fig. S2 in the Supplementary Material). For this reason, a two-filter configuration was used for all subsequent experiments.

Finally, the distal tip of the custom fiber probe was attached to the forefinger of the participant using a 3D-printed finger clip. This finger clip was designed with a spring-loaded mechanism that ensures constant pressure and holds the probe in contact with the skin for the duration of the measurements (thereby minimizing motion artifacts), while providing comfort to the participant. To drive/control the sensor, a custom-written LabVIEW interface controlled all components and provides automatic pre-processing of the collected data. The entire device (including the sensor, fiber probe, finger clip, and laptop running LabVIEW control software) is shown in Fig. 1(d).

### 2.2 Compact Fiber-Optic Fluorescence Sensor—Electronic Configuration

**Fig. 2:**
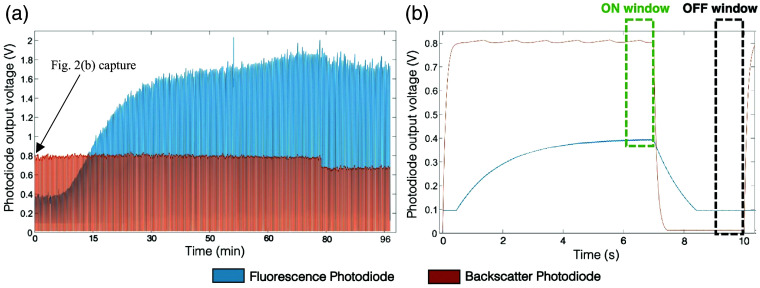
Characteristic signals obtained *in vivo* using the compact fluorescence sensor. (a) Example raw data (after amplification) collected with the compact sensor in a clinical experiment lasting ∼100  min. (b) Close-up of data collected in a single duty cycle (of 10 s) from data displayed in panel (a) (as indicated by the arrow in panel a). Both graphs show signals from both the fluorescence photodiode (blue) and backscatter photodiode (red). ON and OFF window regions are displayed in panel (b). These regions were used to extract the photodiode output voltages for calculation of the normalized fluorescence signal.

The sensor electronics were powered and controlled using an Arduino UNO and a USB data acquisition controller (DAQmx—NI USB-6212, National Instruments), both of which were connected via USB to a laptop computer. The electronic design of the sensor was guided by the input signals detected by the photodiodes. Figure 2 shows an example of a typical raw output signal collected with the sensor in a clinical study participant. As light illuminates the skin, the photodiodes detect the fluorescent and backscattered signals (Fig. 2, blue and red curves, respectively). As the concentration of contrast agent in the bloodstream increases, the fluorescence photodiode signal increases [Fig. 2(a), blue]. Without amplification, the photodiode currents generated by the fluorescence emission are very low (on the order of nA). This implies the need for a high electronic gain on the fluorescence photodiode to produce a measurable output signal. For this reason, gains of 101 and 90 dB were respectively applied to the fluorescence and backscatter photodiodes using two-stage amplification. These gain values were determined via preliminary *in vitro* experiments and computational simulations performed using LTspice. Two-stage amplification was used as the required gain values exceeded those achievable with the amplifier used in the circuit. Gain values were kept constant for all experiments presented herein.

To ensure thermal stability and safe use, a 70% duty cycle (with a 10 s time period) was applied to the LED (i.e., 7 s on and 3 s off), which was used throughout all measurements. This duty cycle was selected based on the characteristics of the detected fluorescence and backscattered signals. As shown in Fig. 2(b), there is a time desynchronization between the fluorescence and backscatter photodiodes. The small currents generated from the fluorescence signal and the corresponding high gain used lead to a delay in the fluorescence photodiode activation response [visible at around 0.5 s; Fig. 2(b)], as well as a slower increase toward a stationary state [plateau stage; Fig. 2(b)]. This is due to slow charging of the photodiode capacitor at the nA current levels generated by the fluorescence signal and the slower amplifier response obtained when using a higher gain. Thus, a 70% duty cycle was selected—with the LED on for 7 s and off for 3 s—to ensure that the plateau stage was reached in both photodiodes. The plateau regions were then used to extract photodiode intensity values for calculation of the normalized fluorescence intensity (see Sec. 2.7).

This duty cycle also ensured that the LED temperature remained constant (thereby avoiding overheating) for the duration of measurements (i.e., when switched on for up to 3 h) and allowed for measurement of normalized transcutaneous fluorescence intensity every 20 s. (An additional wait time of 10 s was incorporated into the LabView control software to allow for processing and to ensure that measurements were made at regular, repeatable intervals. This additional time also allowed for an easier comparison with the benchtop system—see Secs. 2.4 and 2.6.) The LabView interface described above displays both the raw photodiode output voltages [i.e., as shown in Fig. 2(b)] and the normalized fluorescence signal (which is automatically calculated using the plateau regions; Fig. 2(b), on and off windows).

Finally, as discussed above, the backscatter photodiode is used to correct for variations due to parameters such probe orientation/location, LED excitation power, and skin color/thickness. As an example, Fig. 2(a) shows a decrease in the signals from both photodiodes at a time of ∼77  min caused by a change in sensor position. As this is detectable by both photodiodes, the normalized fluorescence signal can be corrected (as it is calculated by dividing the output voltage from the fluorescence photodiode by that from the backscatter photodiode—see Sec. 2.7).

### 2.3 Safety Considerations

To ensure safe use in clinical experiments, the optical power and temperature of the LED were analyzed using an optical power meter (PM100D, Thorlabs) and a thermocouple (MAX31855, Maxim Integrated Products) respectively. Measurements were performed at both the surface of the LED and at the distal tip of the fiber-optic probe for a period of 3 h to investigate the stability of the sensor over the timescale of a typical experiment.

The optical power of the LED at the distal tip of the fiber probe was limited to a maximum allowable power of 63  μW. This was achieved using a constant current LED driver and ensured that the light emitted at the distal end of the fiber-optic probe was always below the maximum permissible exposure for the skin.^18^^,^^19^

### 2.4 Benchtop—Control System

To analyze the performance of the portable fluorescence sensor and to investigate the potential of the device to monitor gut function, an existing benchtop spectrometer was used as a control system. The benchtop system—which was originally reported in Ref. 10—comprises two laser sources (Stradus 488-25 and Stradus 785-80, Vortran Laser Technology, United States) for excitation of fluorescence and a compact spectrometer (FLAME-S-VIS-NIR-ES, Ocean Insight) for detection. Bandpass and neutral density filters are used to clean up the laser emission profiles and to limit the optical excitation power to safe levels. Longpass filters (housed within a motorized filter wheel) are used to reject backscattered excitation light. Finally, a bifurcated optical fiber probe (QR200-7-VIS-NIR, Ocean Insight) is used for light delivery and collection to allow for interrogation of fluorescence signals at the skin. A 488 nm excitation was used for all experiments presented in this article as this provided efficient excitation of fluorescein fluorescence (the fluorescent contrast agent used for all experiments) and was comparable to the excitation wavelength of the compact fluorescence sensor (465 nm).

The benchtop system collects a fluorescence spectrum at every time point and converts the spectrum into a scalar value to represent the fluorescence intensity. This is carried out by summing over the wavelength range of 500 to 580 nm (containing the spectral peak of the fluorescence signal) and then normalizing the integrated fluorescence value according to both integration time and laser power (see further details in Ref. 10). Previous studies using this benchtop system have shown the potential of transcutaneous fluorescence spectroscopy for noninvasive assessment of gut permeability and the gastric emptying rate.^10^^,^^11^^,^^13^ Hence, the benchtop spectrometer represented a suitable comparator system for validation of our novel, compact fluorescence sensor.

### 2.5 Fluorescence Samples Measurement—Laboratory Validation

We first evaluated the sensitivity of the compact fluorescence sensor for the detection of different concentrations of fluorescein in aqueous solution. A series of fluorescent samples (fluorescein solutions) were prepared for this purpose and designed to approximate the fluorescence intensities observed *in vivo* (based on previous data collected using the benchtop system in healthy volunteers). We note that these fluorescent samples were not intended to act as realistic phantoms. Rather, they simply served to provide specific fluorescence intensity levels that corresponded to those observed in vivo, thereby allowing us to determine whether the sensitivity of the compact sensor was sufficient for detecting transcutaneous fluorescein fluorescence. Thus, fluorescein solutions were prepared in water at concentrations of 0  mg/mL (i.e., water with no fluorescein, which represents the baseline), 0.00044, 0.00056, 0.00083, and 0.0017  mg/mL. The fluorescein solutions were contained within transparent thermoplastic pouches for measurements, and fluorescent signals were evaluated using both the compact fluorescence sensor and the benchtop control system (experimental setup is shown in Fig. S3 in the Supplementary Material).

### 2.6 Human Measurement—*In Vivo* Validation

*In vivo* validation of the compact fluorescence sensor was performed in three stages. First, clinical measurements were undertaken in eight healthy volunteers using both devices (i.e., the benchtop system and the compact fluorescence sensor) simultaneously to compare the collected signals (stage 1). Second, additional measurements (using both devices) were performed in six further healthy volunteers using a lower fluorescein dose to demonstrate the potential of the sensor to assess gut function under different dose conditions (stage 2). Third, all 14 healthy volunteers were classified into three groups based on their skin tone (classified according to the Fitzpatrick scale) to determine the performance of the sensor on different skin tones (stage 3). For all measurements, the benchtop sensor was attached to the participant’s index finger and the compact sensor to the participant’s middle finger. The information about participant demographics, as well as the fluorescein dose used in each participant, is shown in Table 1.

**Table 1:** Demographics of the study participants and the respective ingested dose of fluorescent dye. Stage 1: participants 1 to 8; fluorescein dose—500 mg (in 100 mL water). Stage 2: participants 9 to 14; fluorescein dose—200 mg (in 100 mL water). The lower region of the table presents the percentages and numbers of participants in each skin tone and dose group (participant numbers shown in brackets).

ID	Gender (female/male)	Age (years)	Fitzpatrick class (1 to 6)	Fluorescein dose (mg)
P1	F	24	3	500
P2	F	24	1	500
P3	F	24	1	500
P4	M	23	3	500
P5	F	23	4	500
P6	M	23	3	500
P7	M	21	5	500
P8	M	56	3	500
P9	F	24	1	200
P10	M	27	3	200
P11	F	23	1	200
P12	M	37	5	200
P13	F	21	3	200
P14	M	23	3	200
Total participants			14	
Male/female			50%/50%	
Fitzpatrick classes 1 to 2			29% (4)	
Fitzpatrick class 3			50% (7)	
Fitzpatrick classes 4 to 6			21% (3)	
Participants with 500 mg			57% (8)	
Participants with 200 mg			43% (6)	

All volunteers gave informed consent prior to the experiments. All 14 volunteer experiments were performed at St. Mary’s Hospital (London, United Kingdom) according to a local clinical study protocol^20^ (details of ethical approval: UK Health Research Authority IRAS Project ID—242462; Research Ethics Committee reference—18/LO/0714/AM04). All experiments were performed in accordance with Good Clinical Practice guidelines and the World Medical Association’s Declaration of Helsinki.

For stage 1, the fiber probes of the compact fluorescence sensor and the benchtop system were first attached to the participant’s first and middle fingers, respectively. Fluorescence measurements were then started at the same time that the participant was asked to begin drinking a fluorescein solution containing 500 mg fluorescein dissolved in 100 mL water. Fluorescence signals (from the compact sensor) and fluorescence spectra (from the benchtop system) were then recorded for a total of 180 min (with the exception of participants 1 and 2, for whom the experiments were terminated early—after 90 and 135 min, respectively), with normalized fluorescence intensity values (see Sec. 2.7) calculated and reported every 60 and 20 s for the benchtop system and compact sensor, respectively.

For stage 2, both devices (i.e., the benchtop system and the compact fluorescence sensor) were again used simultaneously, this time with a lower dose of fluorescent dye. The fiber-optic probes were attached to the participant’s fingers, and participants were asked to drink a fluorescein solution containing 200 mg of fluorescein dissolved in 100 mL water (as opposed to 500 mg fluorescein used in stage 1). Fluorescence signals were recorded as in stage 1.

For stage 3, all 14 participants were classified into three groups based on their Fitzpatrick skin type. No extra data were collected for this stage. Instead, stage 3 focused on analyzing the collected data to assess the impact of skin tone on the performance of the sensor. First, we analyzed the optical baseline signals (extracted from the first 10 min of data) in which no fluorescence signal from the contrast agent was present. Second, we analyzed the entire fluorescence versus time curves (i.e., 180 min).

In all experimental stages, all volunteers were seated for the duration of the experiments and were asked to fast overnight prior to the experiments.

### 2.7 Calculation of Normalized Fluorescence Values

To account for signal variations due to factors such as excitation power fluctuations, probe orientation, and skin tone, we normalized the collected fluorescence data according to the intensity of the backscattered excitation signal. This normalization was performed for data collected with both the compact sensor and the benchtop system.

For the benchtop system, the normalization process was conducted as described in Ref. 10. For the compact sensor, a preprocessing step was implemented in the software to first subtract the baseline from the raw data collected in each LED duty cycle (for both the fluorescence and backscatter photodiodes). This was achieved by calculating the average intensity value over a period when the LED was switched off [i.e., during the OFF window shown in Fig. 2(b)] and subtracting this value from the raw data at each time point. Fluorescence and backscattered intensity values were then calculated (based on the baseline-subtracted signals) by taking the average photodiode signals over a period when the LED was switched on and the observed signals had reached a plateau [i.e., during the ON window shown in Fig. 2(b)]. A normalized fluorescence intensity value was then calculated for each LED duty cycle according to Eq. (1), where $I_{\text{norm}}$ is the final normalized fluorescence intensity; $I_{F}$ and $I_{L}$ are the average fluorescence and backscattered intensity values, respectively [i.e., values from the ON window in Fig. 2(b)]; and $I_{\mathrm{BGF}}$ and $I_{\mathrm{BGL}}$ are the average background fluorescence and backscattered intensity, respectively [i.e., values from the OFF window in Fig. 2(b)]:

$$I_{\text{norm}}=\frac{\left(I_{F}-I_{\mathrm{BGF}}\right)}{\left(I_{L}-I_{\mathrm{BGL}}\right)}.$$ (1)

As explained above, this normalization procedure generated fluorescence intensity values for the compact sensor and benchtop system that were internally corrected to account for variations in excitation power, probe orientation, and other factors. Nonetheless, the absolute values produced in each case were different across the two devices (due to differences in the detection electronics used and the corresponding outputs—i.e., the photodiodes in the compact sensor provided voltage output values, and the spectrometer in the benchtop system provided intensity measurements in arbitrary spectral units). To allow for comparison of the datasets collected with each system, we scaled the collected fluorescence versus time curves to their maximum intensity. This meant that the collected fluorescence versus time curves had an intensity range of 0 to 1 for both the compact sensor and the benchtop system, thereby allowing for comparison of the datasets and investigation of the degree of correlation. Importantly, 0 to 1 scaling was only applied when comparing devices (i.e., in experimental stage 1—see Sec. 2.6) and not when investigating the impact of dose and skin tone (i.e., stages 2 and 3).

### 2.8 Data Analysis

Statistical tests were performed to analyze the correlation/differences between devices (stage 1) and to assess the performance of the compact sensor under different experimental conditions (stages 2 and 3). Pearson’s correlation coefficient (r) was calculated to measure the linear correlation between datasets collected with the compact fluorescence sensor and benchtop system (using the scaled fluorescence signals for both individual and mean fluorescence versus time curves). P<0.05 were used to infer statistically significant correlations. Pearson’s correlation coefficients (r) were calculated using the “corrcoef” MATLAB function.

Student’s t-tests were used to analyze differences between data collected with each device (stage 1, paired t-test) and data collected under different dose and skin tone conditions (stages 2 and 3, unpaired t-test). For this purpose, the peak intensity, peak time, total area under the curve (AUC), and AUC up until the time of the peak (peak AUC) were extracted from each dataset and compared across groups (with scaled fluorescence signals (0 to 1) used for stage 1 and normalized fluorescence signals used for stages 2 and 3). These parameters were chosen to provide a simple characterization of the shape and intensity of the fluorescence versus time curves collected with the two devices (e.g., the total AUC parameter provides a characterization of the total fluorescence intensity, the peak time provides an indication of the rate of uptake of fluorescein, etc.). Thus, these parameters served to allow for an effective assessment and comparison of the data collected with different devices and under different conditions.

## 3 Results and Discussion

### 3.1 Laboratory Validation

#### 3.1.1 Fluorescent samples measurements

To validate the compact fluorescence sensor, we first assessed the capability to detect fluorescein fluorescence in aqueous solutions with different known dye concentrations. The fluorescein concentrations used were specifically selected to approximate transcutaneous signal levels expected in healthy participants. To this end, the fluorescein concentrations were calculated based on transcutaneous data previously collected from healthy participants (i.e., as reported in Ref. 10). Participants drank a solution of 500 mg fluorescein in 100 mg water, and the benchtop system^10^ recorded the fluorescence signals for 3 h. The peak intensities observed in those transcutaneous datasets were then compared against fluorescence signals collected (using the benchtop system) from a series of aqueous fluorescein solutions with known concentrations. Based on these measurements, we were able to pair the transcutaneous signal with an approximate corresponding dye concentration in aqueous solution. The chosen aqueous fluorescein concentrations for onward experiments were thus in the range 0 to 0.0017  mg/mL as this range was found to approximate the varying level of fluorescein fluorescence detected during transcutaneous experiments.

Laser welded^21^ thermoplastic containers were fabricated and filled with the chosen fluorescein solutions. A nonfluorescent solution (i.e., water; dye concentration = 0 mg/mL) was also measured to assess any background signals (e.g., from the encapsulation material).

**Fig 3:**
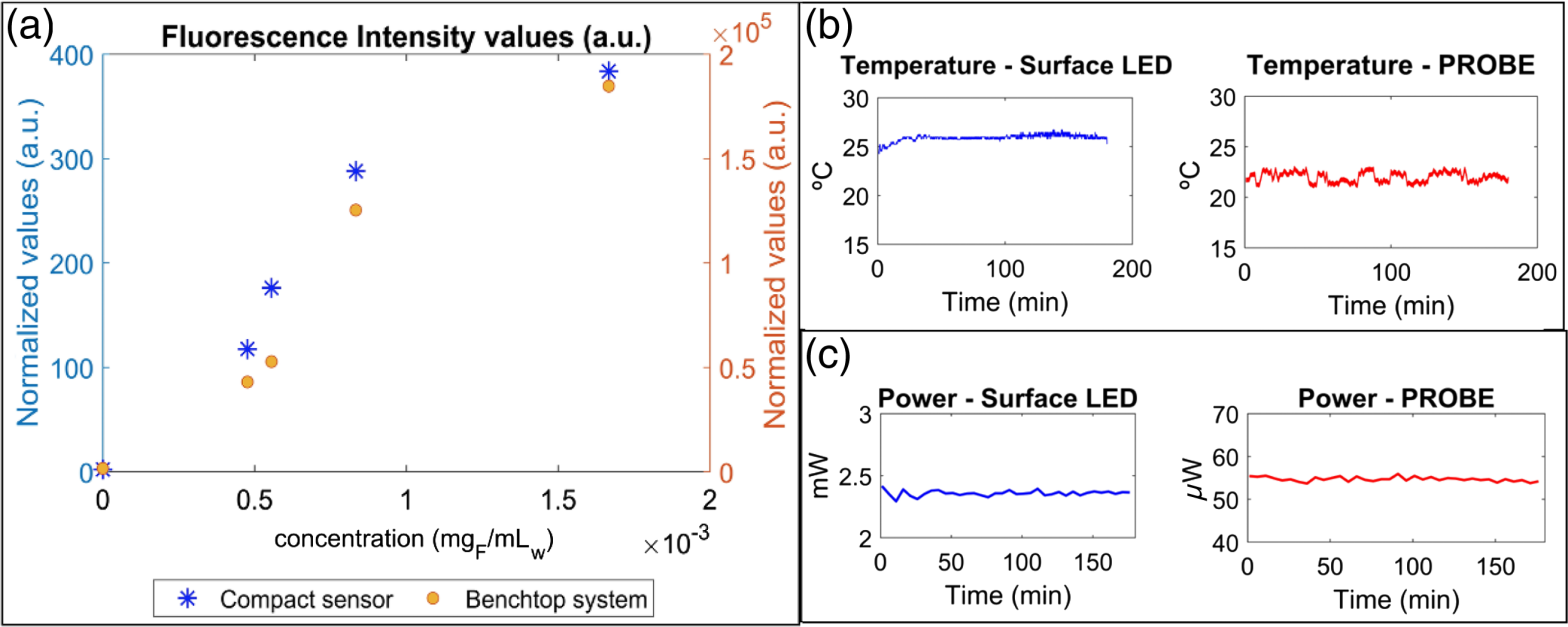
Laboratory validation of the compact fluorescence sensor (against benchtop system) and safety evaluation. (a) Normalized fluorescence intensity as a function of fluorescein concentration (in aqueous solutions) for the compact sensor (blue, left y-axis) and benchtop system (orange, right y-axis). Concentration range: 0 to 0.0017  mg/mL. Baseline levels (signals from a concentration of 0  mg/mL fluorescein) for each system were subtracted from all measurements (hence, the first data point occurs at the origin (0,0) for both devices). (b) Temperature measurements collected over a time period of 3 h for two different locations: surface of the LED (left) and tip of the fiber probe (right). Temperature measurements (at the surface of the LED) over three continuous duty cycles are presented in Fig. S4 in the Supplementary Material. (c) Optical power measurements collected over a time period of 3 h for the same locations: surface of the LED (left) and tip of the fiber probe (right). The background optical power (from ambient light) was measured and subtracted from the recorded signals.

Following preparation of the fluorescein solutions, fluorescence measurements were performed using both the compact sensor and benchtop system. Fluorescence spectra peak intensities from the benchtop system were compared against output voltages from the compact sensor, and similar trends (with respect to fluorescein concentration) were observed for both devices [Fig. 3(a)]. A Pearson’s correlation coefficient of r=0.983 was obtained when comparing the two datasets, with a p-value of 0.003. In addition, a linear regression model (i.e., for the equation y=mx+c, where m represents the gradient and c represents the intercept) was fit to both datasets. For the benchtop data, the linear regression yielded $R^{2}=0.944$ (p=0.005) with $m=1.15\times 10^{8}\;{\left(\mathrm{mg}/\mathrm{mL}\right)}^{-1}$ (units = [$\frac{\text{normalized fluorescence intensity}}{\text{fluorescein concentration}}$]) and c=178 a.u. (units = [normalized fluorescence intensity]). For the compact sensor data, the linear regression model yielded $m=2.31\times 10^{5}\;{\left(\mathrm{mg}/\mathrm{mL}\right)}^{-1}$ and c=30.3 a.u., with an $R^{2}$ value of 0.922 (p=0.009). These results demonstrate strong (statistically significant) linear correlations between the fluorescence intensity and fluorescein concentrations for both the benchtop and compact sensors. Taken together, these observations suggested that the compact fluorescence sensor was capable of detecting fluorescein fluorescence with signal levels that would be expected *in vivo*.

#### 3.1.2 Temperature and optical power measurements

To confirm that the compact fluorescence sensor was safe for use in clinical experiments, temperature and optical power measurements were performed for 3 h (i.e., the duration of a typical clinical experiment) using a thermocouple and an optical power meter. Two locations were investigated: the surface of the LED and the tip of the fiber probe. The temperature was stable over time at both locations [Fig. 3(b)] and was also stable over individual LED duty cycles (Fig. S4 in the Supplementary Material). Furthermore, the average temperatures measured at the LED and the fiber tip (25.9 and 22.0°C, respectively) were found to lie within a safe range (i.e., close to room temperature), indicating that the compact sensor would not expose participants to unpleasant or dangerous temperature conditions.

Similarly, the optical power exhibited only minor variations over the duration of the experiment [at both the LED surface and fiber tip; see Fig. 3(c)], indicating stability of the light source current provided by the LED driver. The average optical power at the tip of the fiber probe was measured as 54.7  μW, below the maximum allowable power of 63  μW (which was calculated according to the guidelines presented in Refs. 18 and 19).

### 3.2 *In Vivo* Validation

#### 3.2.1 Validation of compact fluorescence sensor against benchtop system—stage 1

To investigate the potential of the compact fluorescence sensor as a portable tool for noninvasive assessment of gut function using transcutaneous fluorescence spectroscopy, we compared the performance against an existing benchtop system (presented in Refs. 10, 11, and 13). To do so, eight healthy volunteers (participants 1 to 8) were recruited for an *in vivo* validation experiment in which the compact sensor and benchtop system were used to record the fluorescence profiles simultaneously. As the benchtop system has previously been shown to be suitable for noninvasive sensing of both gut permeability^10^^,^^13^ and the gastric emptying rate,^11^ validation against this system served to demonstrate the potential of the compact sensor for the same purpose with the advantages of a smaller footprint and lower cost.

**Fig 4:**
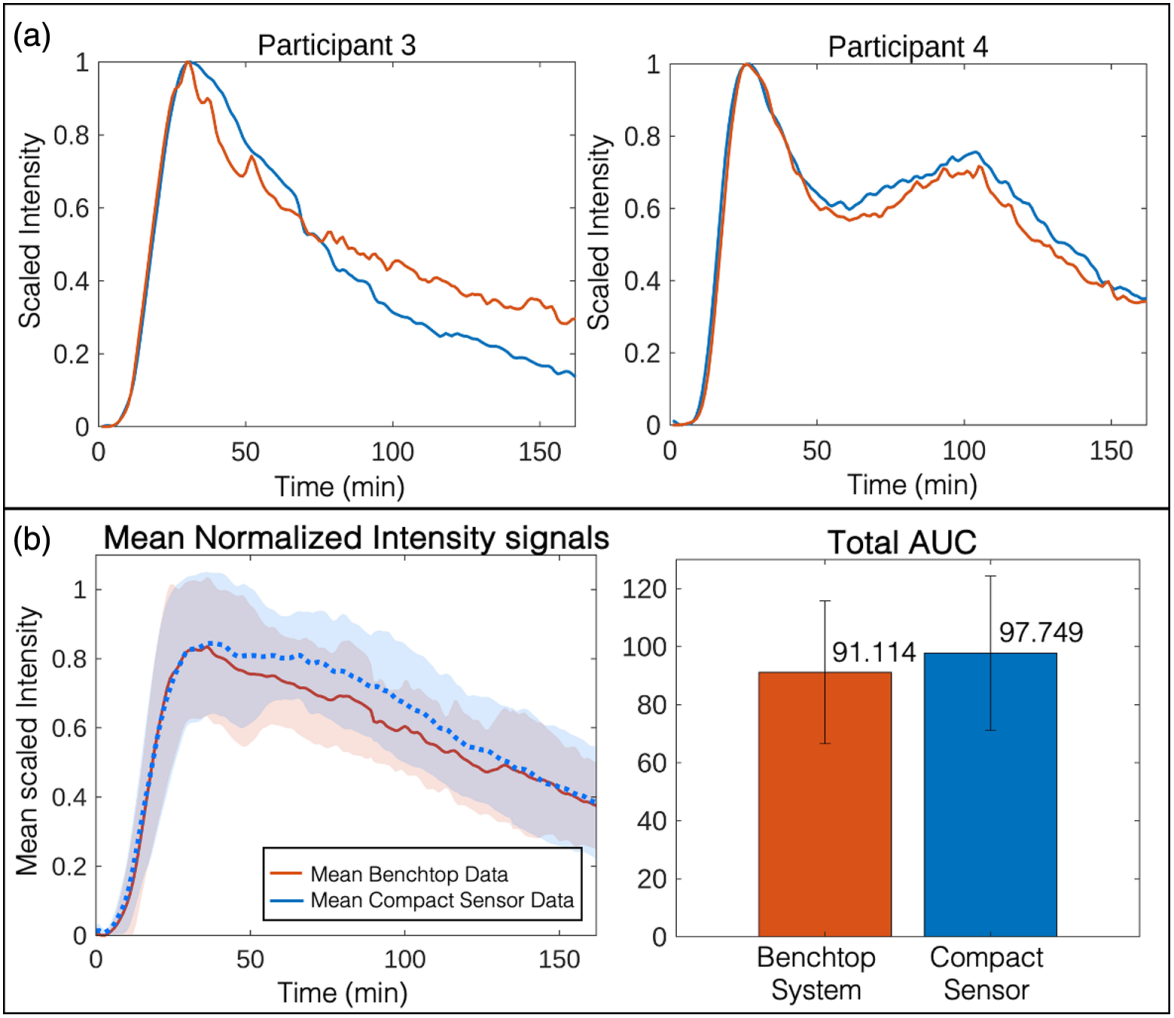
*In vivo* validation measurements in healthy volunteers. (a). Examples of scaled fluorescence intensity versus time curves from compact fluorescence sensor (blue) and benchtop system (orange) for participants 3 and 4. Participant 3 shows a typical time-resolved fluorescence curve, with a single peak followed by a decay toward the baseline. Participant 4, on the other hand, exhibits a “double peak phenomenon” (an effect observed in certain drugs^22^ with biphasic absorption behavior). (b) The mean and STD of the fluorescence intensity versus time curves for both the compact sensor (blue) and the benchtop system (orange). The individual scaled fluorescence intensity versus time curves for participants 1 to 8 are presented in Fig. S5 in the Supplementary Material. (c) The mean AUC calculated for both systems (for participants 1 to 8). Error bars represent upper and lower bounds on the AUC values calculated by assessing the AUC for the mean curves ±1 STD (i.e., the AUC values corresponding to the upper and lower bounds of the shaded regions in panel (b). Total AUC values for each participant and for the mean curves are presented in Table S2 in the Supplementary Material.

The eight participants were asked to fast for a minimum of 5 h prior to the experiment. At the start of the measurement, the fiber-optic probes from the compact sensor and the benchtop system were attached to the participant’s fingers using 3D-printed finger clips. Participants were asked to drink a fluorescein solution (500 mg fluorescein dissolved in 100 mL water), and fluorescence data were collected with both devices for 180 min. Fluorescence intensity versus time curves (scaled from 0 to 1) from both systems were plotted to compare the two datasets (Fig. 4). As expected, fluorescence intensity was observed to increase over time, reaching a peak value ∼40  min after ingestion of the fluorescein solution, before beginning to decrease back toward the background level (Fig. 4). We note that small differences in the decay rate were observed between the benchtop system and the compact sensor in some participants (e.g., see participants 3, 6, and 8; Fig. S5 in the Supplementary Material). These can be tentatively attributed to variations in vascular anatomy at the measurement sites (as the benchtop and compact systems were connected to different fingers); motion artifacts or probe movement; and/or probe placement differences. Despite these small discrepancies in the decay region, good qualitative agreement was nonetheless observed between the compact fluorescence sensor data and the benchtop data, both in all eight participants individually [Fig. 4(a) and Fig. S5 in the Supplementary Material] and in the mean fluorescence versus time curves [Fig. 4(b)].

To quantify the correlation between the datasets, Pearson’s correlation coefficients were calculated for all individual measurements (presented in Fig. S5 in the Supplementary Material) and the average curves [Fig. 4(b)]. As shown in Table S1 in the Supplementary Material, all Pearson’s correlation coefficients (r) exceed 0.919 (with p<0.001 in all cases).

To further assess the similarity between the datasets, we compared the total AUC [and the respective standard deviations (STD)] of all individual curves (Fig. S5 in the Supplementary Material) as well as the average fluorescence intensity curves for both systems [Fig. 4(b)]. As shown in Fig. 4(c), both datasets exhibited similar AUC values, with a difference of 6.787% (which was not statistically significant, as determined by a paired Student’s t-test—p=0.142). Total AUC values and other extracted parameters (such as peak AUC and peak time) for all individual signals, as well as the statistical analysis results, are presented in Tables S2 and S3 in the Supplementary Material. Overall, this demonstrated that the performance of the compact fluorescence sensor was comparable to the clinically validated benchtop system, thereby indicating its potential for clinical assessment of gut function.

As an aside, it is worth noting that the range normalization approach used in this section (i.e., scaling data from 0 to 1) would not be suitable for the investigation of intestinal permeability (for which it is necessary to assess changes in fluorescence intensity between participants, e.g., as described in Ref. 13). Instead, this approach served to allow for comparison and validation of the compact sensor against an existing system that has been deployed in clinical studies, thereby demonstrating the development of a more affordable and portable technology that is better suited to large-scale clinical deployment.

#### 3.2.2 Compact sensor characterization—impact of contrast agent dose—stage 2

Following validation against the benchtop device, a second experimental stage was introduced to characterize the response of the compact fluorescence sensor using a lower fluorescein dose. To do so, six further healthy volunteers (participants 9 to 14) were recruited following the same experimental protocol described above but with a lower fluorescein dose (200 mg fluorescein in 100 mL water).

**Fig 5:**
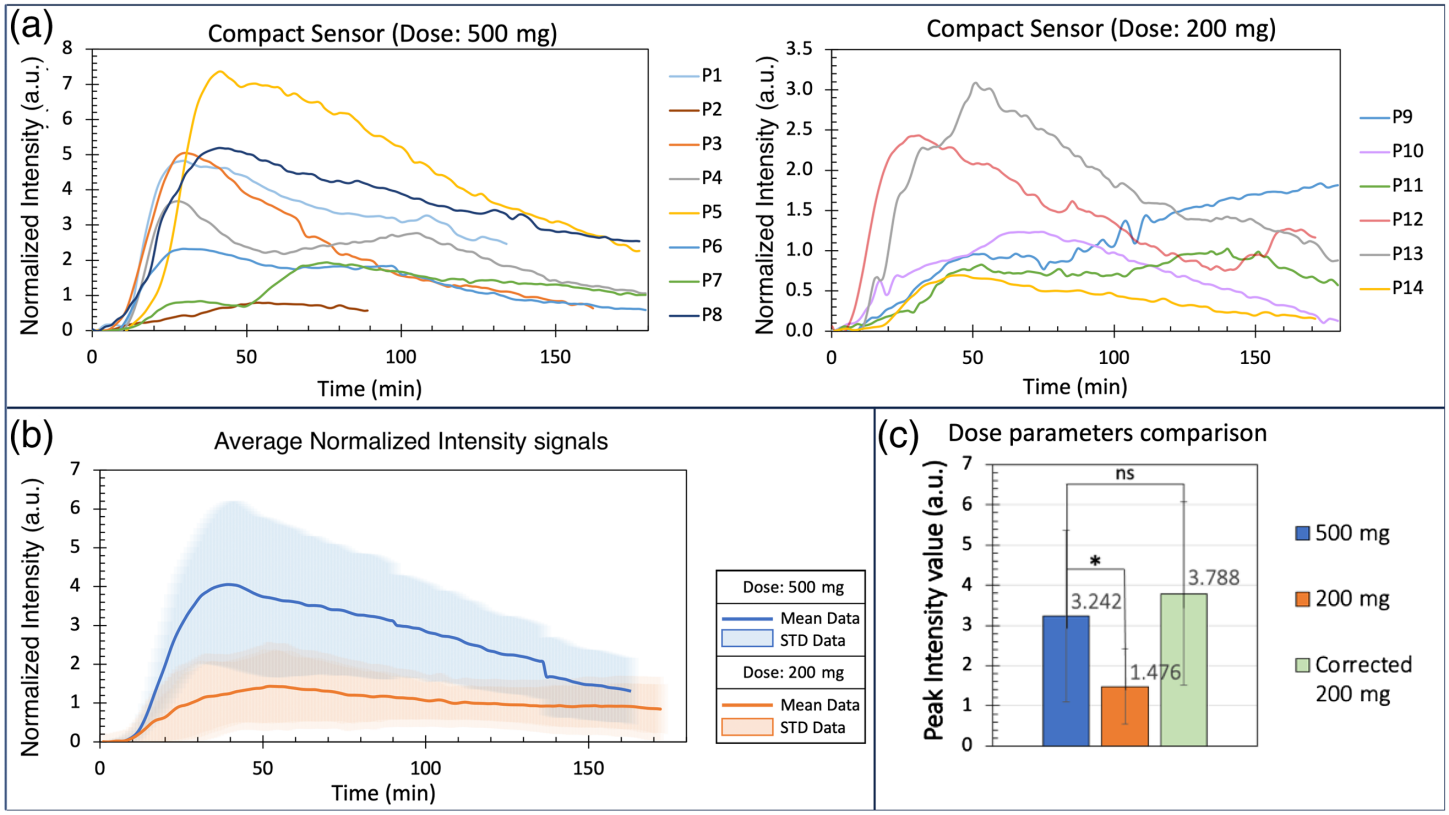
Characterization of compact sensor under different fluorescein dose conditions. (a) Time-resolved normalized fluorescence signals for participants 1 to 8 (left; 500 mg fluorescein in 100 mL water) and participants 9 to 14 (right; 200 mg fluorescein in 100 mL water). Participants 1 and 2 present shorter signals (135 and 90 min, respectively) due to data collection issues that necessitated early termination of experiments. (b) Mean (±STD) normalized fluorescence versus time curves for both dose conditions: 500 mg (blue) and 200 mg (orange). The irregularities/discontinuities observed at 90 and 135 min on the mean normalized fluorescence signal for the 500 mg dose are a result of the shorter data collection times for participants 1 and 2. (c) Mean peak (maximum) intensity values extracted from the normalized intensity curves shown in panel (a). Note that the maximum intensity values in panel (c) do not correlate to the maximum values observed on the curves shown in panel (b) due to variability in the time at which peak intensities were observed in each participant [see panel (a)], that is, the values reported in panel (c) represent the averages of the maximum intensity values observed in each curve in panel (a). Thus, any effect of peak time is removed/ignored in the average calculation in panel (c) [unlike in panel (b), in which varying the peak time leads to changes in the location and intensity of the curve’s peak]. A statistically significant difference was observed between the 500 and 200 mg peak intensity values. When a correction factor (of 2.5) was applied to the 200 mg peak intensity (“corrected 200 mg”; green bar), no statistically significant difference was observed when compared against the 500 mg dose, indicating an approximately linear relationship between dose and peak intensity. *—p<0.05; ns, not significant.

Figure 5(a) shows the fluorescence versus time curves for all 14 participants (left—participants 1 to 8, 500 mg fluorescein; right—participants 9 to 14, 200 mg fluorescein). In this case, normalized fluorescence intensity signals are presented (as opposed to the scaled signals used in Sec. 3.2.1) to allow for the quantification of changes in fluorescence intensity with respect to dose. As observed in Fig. 5(a), most of the datasets exhibit an increase in fluorescence intensity up to a peak point prior to a decay back toward zero. A similar trend is observed in the mean fluorescence intensity versus time curves for both dose conditions [Fig. 5(b)].

The only exception to this trend is the data collected in participant 9, in which no decay region is observed and peak intensity appears to occur at ∼180  min (i.e., at the end of the 3 h acquisition). Importantly, the dynamics observed for participant 9 were the same with both the compact sensor [Fig. 5(a)] and the benchtop system (Fig. S7(a) in the Supplementary Material). Thus, this behavior can be attributed to physiological factors intrinsic to the participant (e.g., slow gastric emptying, low GI motility, etc.).

The maximum fluorescence intensity observed for the 500 mg dose (4.053 a.u.) was higher than that observed for the 200 mg dose (1.433 a.u.) by a factor of 2.8 [Figs. 5(b) and 5(c); also see Fig. S6 in the Supplementary Material]. This difference was found to be statistically significant using an unpaired Student’s t-test (p=0.0371). Importantly, this significant difference is compensated when a correction factor of 2.5 is applied to the 200 mg dose signals (p=0.741), suggesting an approximately linear correlation between dose and fluorescence intensity (i.e., indicating that a 2.5-fold increase in dose produces pprox.rox. 2.5-fold increase in intensity). Results obtained from analysis of the total AUC and analysis of equivalent data collected with the benchtop system (collected at the same time as the data presented in Fig. 5) further support this hypothesis (see Figs. S7–S9 in the Supplementary Material).

#### 3.2.3 Compact sensor characterization—impact of skin tone—stage 3

The final experimental stage (stage 3) aimed to characterize the response of the compact fluorescence sensor when applied to different skin tones. All 14 participants were classified into three groups based on their Fitzpatrick skin tone score (i.e., Fitzpatrick type 1, type 3, or type 4/5). Photographs of the hands and arms of three participants representing Fitzpatrick types 1, 3, and 5 are shown in Fig. S10(a) in the Supplementary Material.

**Fig. 6:**
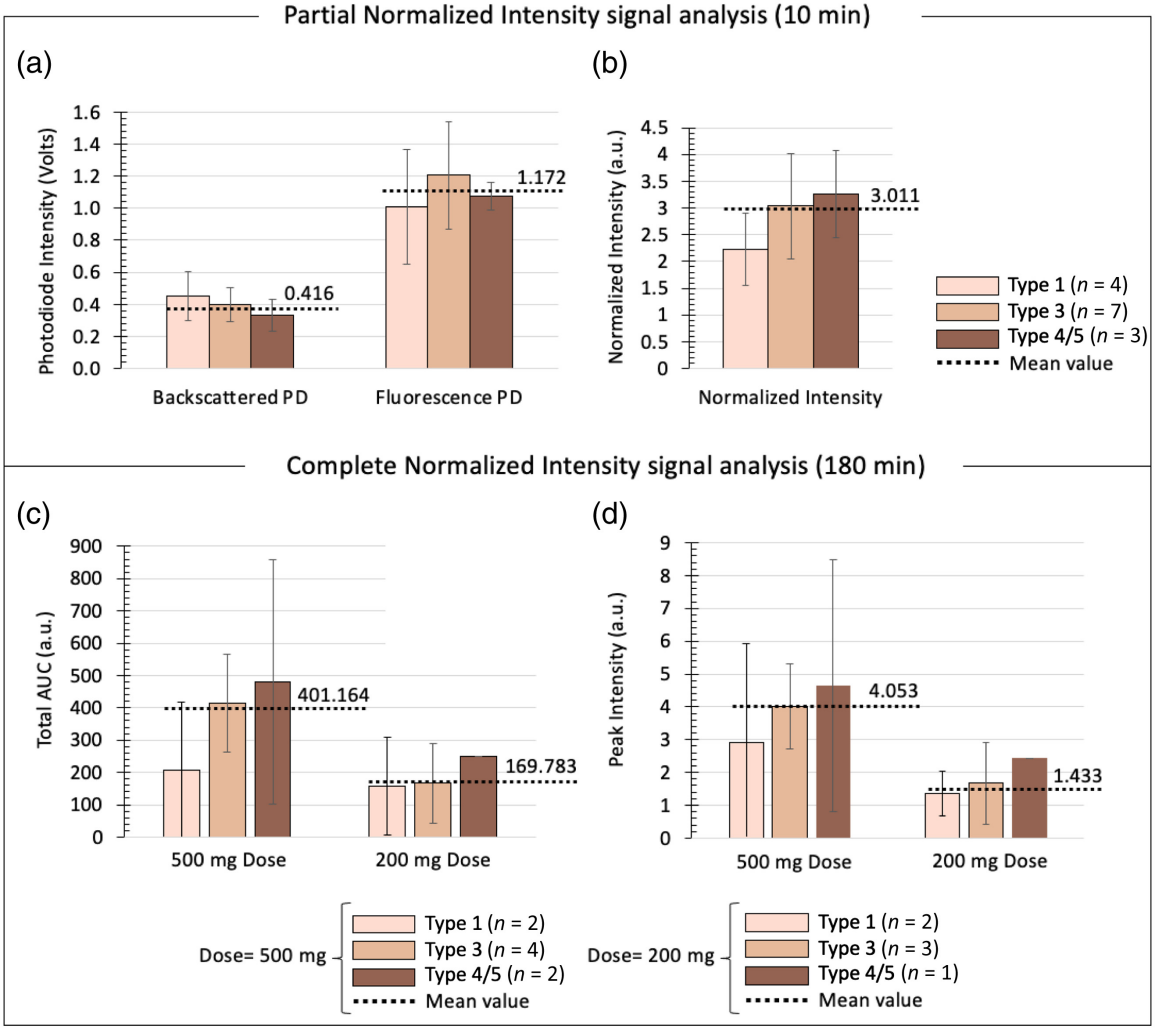
Characterization of compact sensor under different skin tone conditions. (a) Mean (±STD) backscattered and fluorescence photodiode values from the first 10 min of the collected data (i.e., in which no fluorescein signal is present) for each Fitzpatrick group (i.e., types 1, 3, and 4/5). (b) Mean (±STD) normalized intensity values extracted from the first 10 min of data for each Fitzpatrick group. (c) Mean (±STD) total AUC values extracted from the full normalized fluorescence versus time curves (180 min) for each Fitzpatrick group and each dose. Note that no error bar is shown for type 4/5, 200 mg dose, as only one participant was categorized into this group. (d) Mean (±STD) peak intensity values extracted from the full normalized fluorescence versus time curves (180 min) for each Fitzpatrick group and each dose. Bars are color-coded according to Fitzpatrick skin tone type (see legends). The numbers of participants included in each group (including dose conditions) are shown in the legends (total number of participants: n=14). Dotted lines represent the mean values extracted from the curves averaged across all participants (i.e., from the curves presented in Fig. 5(b) for the normalized intensity values), in which Fitzpatrick classification is not considered.

First, we analyzed the first 10 min of the data collected in each participant (in which fluorescein signals are negligible due to the time delay in fluorescein reaching the blood stream following ingestion). Figures 6(a) and 6(b) show the mean values extracted from the fluorescence and backscattered photodiodes [Fig. 6(a)] and the mean normalized intensities [Fig. 6(b)] for each Fitzpatrick group (types 1, 3, and 4/5). Individual values for each participant are shown in Fig. S10 in the Supplementary Material. Only minimal (nonsignificant) differences are observed between Fitzpatrick groups for all parameters (p>0.05 in all cases). However, as expected, the lightest skin type (type 1) and the darkest skin type (type 4/5) present the highest and lowest mean backscattered photodiode values (0.451 and 0.329, respectively).

Second, we analyzed the entire datasets (180 min) in which the overall fluorescence signal is mainly attributed to fluorescein. We extracted the total AUC and the peak intensity values from the signals presented in Figs. 5(a) and 5(b) and calculated the mean values (and STDs) for each Fitzpatrick group [Figs. 6(c) and 6(d)]; individual participant values color-coded by their Fitzpatrick type are presented in Figs. S6 and S8 in the Supplementary Material for the compact sensor and benchtop system, respectively). As above, only minimal, nonsignificant differences (p>0.05) are observed between Fitzpatrick groups (for both dose conditions).

Taken together, these results suggest that the performance of the compact sensor is not adversely affected by changes in skin tone and indicate that the normalization procedures used (i.e., the backscattered intensity is used to correct the fluorescence signal level) provide an adequate correction for any changes in fluorescence intensity observed across different tones.

Overall, our results demonstrate strong correlation of data collected with our compact fluorescence sensor with data from an existing benchtop system.^10^ This indicates the potential of our compact sensor to transcutaneously assess gut function in a more affordable and portable manner than previously reported. Further characterization of our compact sensor suggests a linear relationship between fluorescein dose and fluorescence signal, exhibiting strong signal-to-noise ratios at fluorescein doses of 200 mg. Importantly, the standard, approved dose of fluorescein used in the clinic is 500 mg (e.g., in fluorescein angiography, in which up to 5 mL of fluorescein solution is intravenously injected at a concentration of 100  mg/mL).^23^^,^^24^ Thus, although the use of lower contrast agent doses has the potential to introduce fluctuations in the fluorescence intensity as a result of lower signal-to-noise ratios, our results suggest opportunities to perform transcutaneous spectroscopy using lower fluorescein doses than those typically used in the clinic (i.e., at 200 mg or below). Furthermore, analysis of data with respect to skin tone revealed that variations in skin tone had no discernible effect on the performance of our compact sensor. The ability to detect fluorescence signals under different skin tone conditions and fluorescein doses indicates clear benefits in terms of cost, safety, and patient experience and suggests the potential to successfully deploy transcutaneous spectroscopy in diverse populations and ethnicities. As such, our ongoing and future work now involves deploying transcutaneous spectroscopy in clinical studies of IBD and undernutrition (see preliminary results reported in Ref. 25) to further investigate the potential for non-invasive assessment of gut function.

## 4 Conclusions

We have presented a compact fluorescence sensor for noninvasive assessment of gut function. This device exhibited agreement with a benchtop spectrometer (previously described in Ref. 10) while providing clear advantages in terms of cost and portability. We analyzed the performance of our compact sensor under different contrast agent doses, suggesting a linear relationship between the ingested dose and the observed fluorescence intensity. In addition, we characterized the sensor’s response when applied to different skin tones, with results indicating a minimal impact of skin tone on sensor performance. Overall, our results demonstrate the potential of our compact, transcutaneous fluorescence sensor to provide noninvasive assessment of gut function and to facilitate clinical uptake of transcutaneous spectroscopy on a larger scale.

## Supplementary Material



## Data Availability

Data underlying the results presented in this paper are not publicly available at this time but may be obtained from the authors upon reasonable request.
