# Supplementary material for: Transcutaneous fluorescence spectroscopy: development and characterization of a compact, portable, and fiber-optic sensor
Source: J Biomed Opt. 2024 Feb 28;29(2):027003. doi: 10.1117/1.JBO.29.2.027003 (PMC10900991; doi:10.1117/1.JBO.29.2.027003)
Supplement: Supplementary file 1 [file JBO_029_027003_SD001.pdf]

SUPPLEMENTARY INFORMATION

Transcutaneous fluorescence spectroscopy: development and characterization of a compact, portable, fiber-optic sensor

Elena Monfort Sanchez[1,2]*, James Avery[1,2], Jonathan Gan[2], Jingjing Qian[1], Nilanjan Mandal[1,2], Arjun Agarwal[2], Mulima Mwiinga[3], Rose Banda[3], Ara Darzi[1, 2], Paul Kelly [3,4], Alex J. Thompson[1,2]*

[1]The Hamlyn Centre, Institute of Global Health Innovation, South Kensington, Imperial College London, SW7 2AZ, UK
[2] Department of Surgery & Cancer, St. Mary's Hospital Campus, Imperial College London, W2 1NY, UK
[3]Tropical Gastroenterology and Nutrition Group, University of Zambia School of Medicine, Lusaka, Zambia
[4]Blizard Institute, Queen Mary University of London, London, E1 2AT, UK

** e.monfort-sanchez19@imperial.ac.uk; alex.thompson08@imperial.ac.uk*

This PDF file includes:

Supplementary Figures S1-S10

Supplementary Tables S1-S3

(A)

(B)

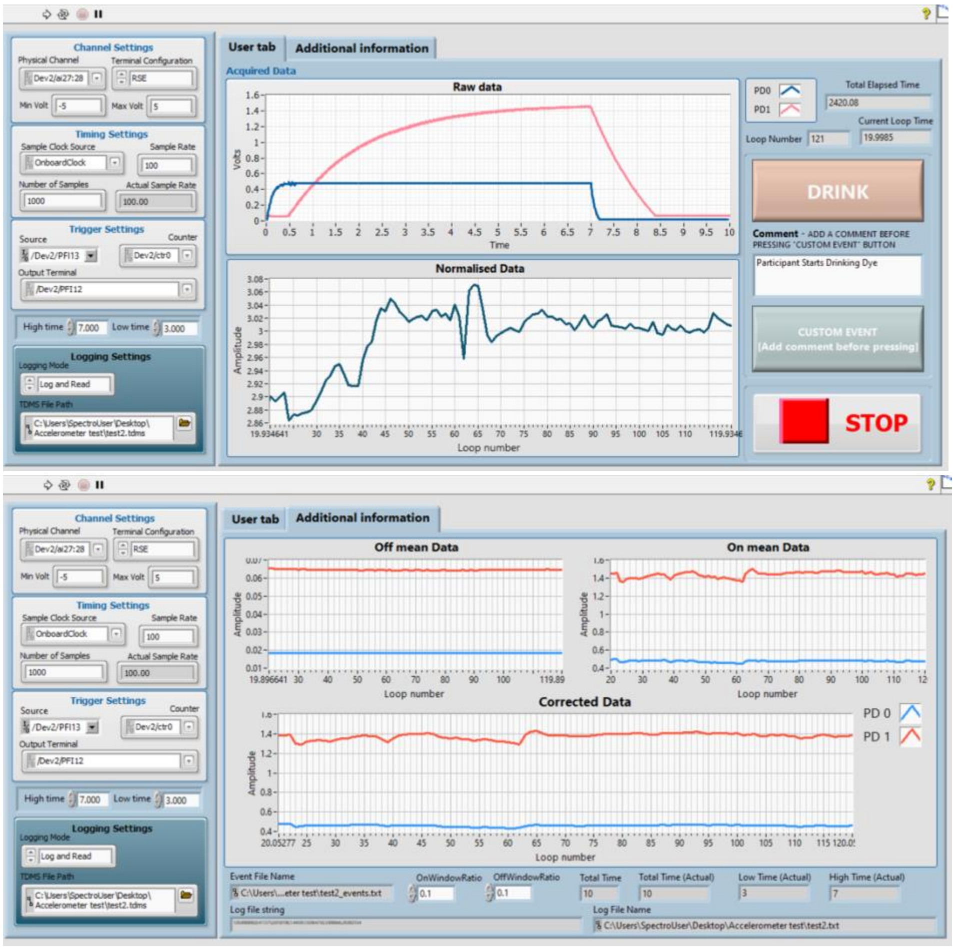

Fig S1. The LabView user interface developed for collection and pre-processing of data. **(A)** Main tab of the LabView interface showing the raw voltage data collected by the sensor photodiodes (upper graph; PD0 – Backscatter Photodiode; PD1 – Fluorescence Photodiode), the normalized fluorescence intensity data (lower graph), and the main sensor/acquisition controls. **(B)** Secondary interface tab with additional information (including mean 'Off' and 'On' data for each photodiode).

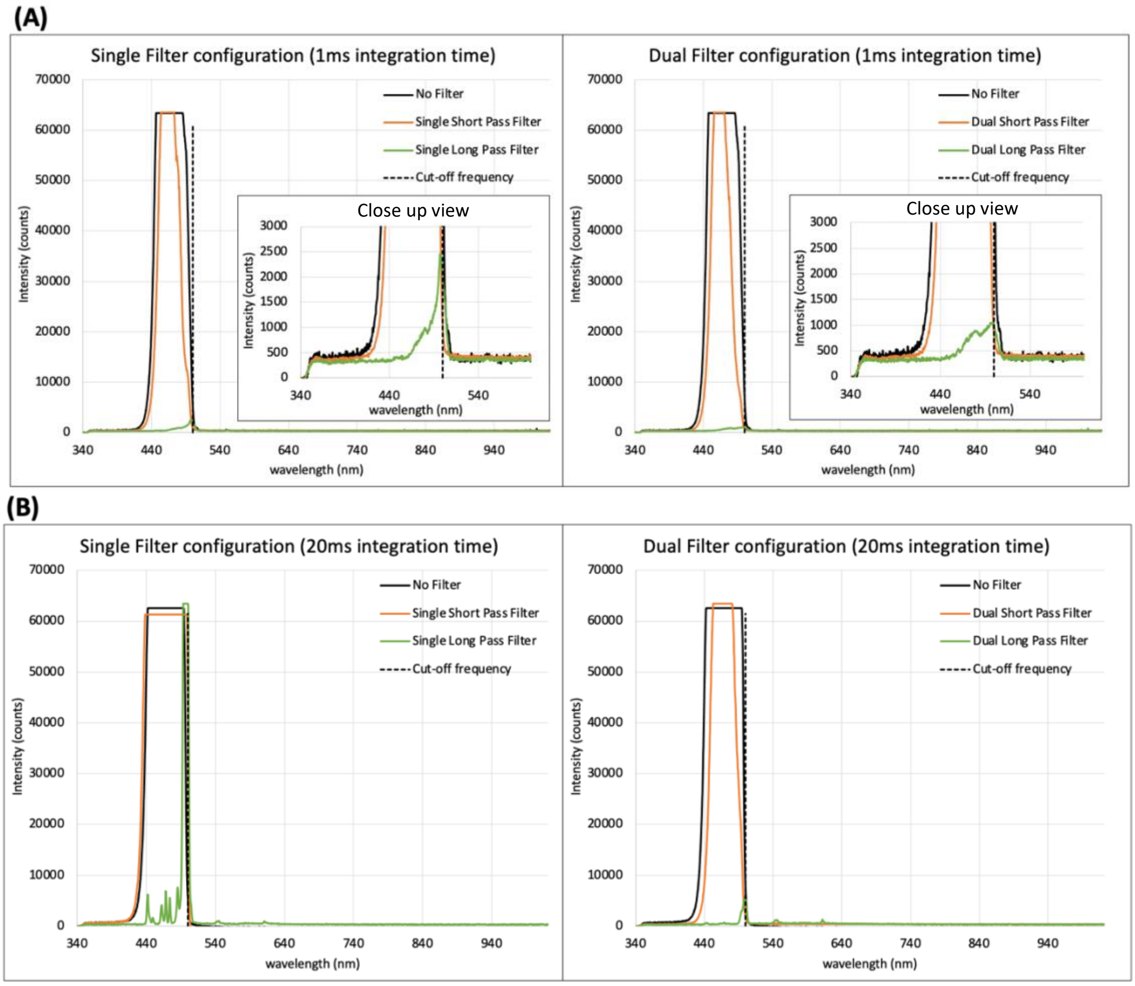

Fig. S2. Light suppression comparison between single and dual filter configuration. Graphs show spectra collected using a FLAME spectrometer (Ocean Insight) when LED excitation light (465 nm central emission wavelength) was directed through long pass and short pass filters (with cut-off frequency at 500 nm) into an optical fiber probe connected to the spectrometer. (**A**) Spectra acquired for single (*left*) and dual (*right*) filter configurations with 1 ms integration time. Inset graphs show close-up views of the spectra. (**B**) Spectra acquired for single (*left*) and dual (*right*) filter configurations with 20 ms integration time. For both integration times, the residual LED signal is considerably lower when using the dual filter configuration as compared to the single filter configuration.

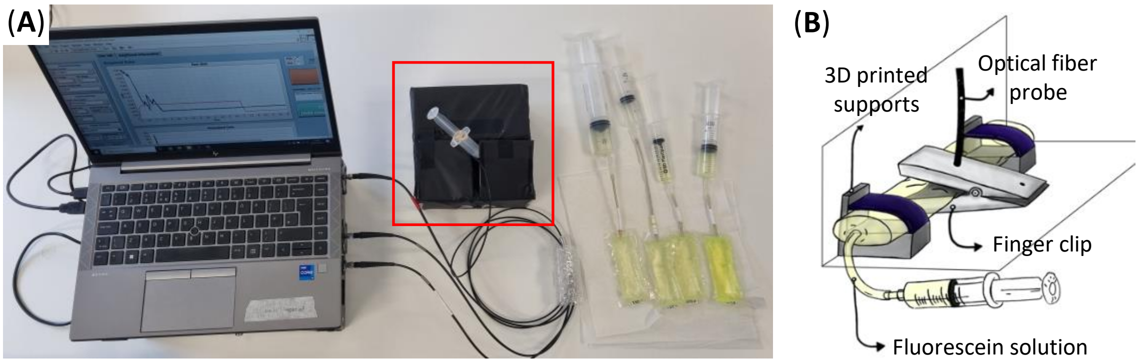

Fig S3. *In vitro* experimental setup. **(A)** Photograph of experimental setup. The sensor (placed below the laptop) collects the fluorescence intensity of fluorescein solutions in transparent thermosplastic containers using the 3D printed finger clip. Thermoplastic containers are displayed on the right-hand side of the image and contain aqueous fluorescein solutions at concentrations of 0-0.0017 mg/ml (with a total volume of 25 ml of fluorescein solution in each thermoplastic container). Container with 0 mg/ml fluorescein (i.e., baseline containing only water) is not visible in the image. This container is located inside a black box (marked in red), which was used to minimize background signals. (**B**) Diagram of the inside of the black box (marked in red in panel **A**) used to collect fluorescence data. The fluorescein solution is attached to the inside of the box using 3D printed supports to maintain a constant position during measurements. The finger clip (including the optical fiber probe) is clamped to the fluorescein sample. The spring-loaded mechanism of the finger clip allows a secure and constant position to be maintained throughout the collection time.

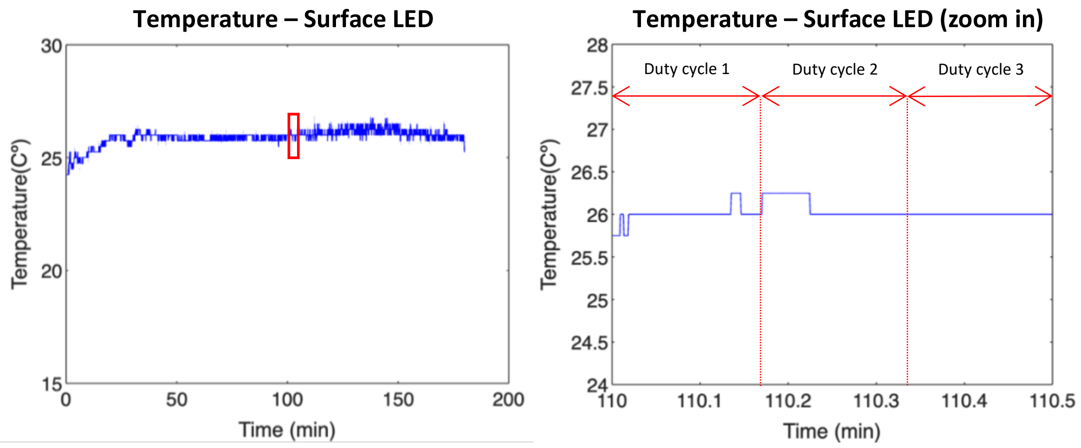

Fig S4. Temperature measurement at the surface of the LED. **(A)** Temperature as a function of time for data collected over a time period of 3 hours (180 minutes). **(B)** Close up of the temperature vs. time data for the region highlighted by the red box in (A). The period displayed represents three complete LED duty cycles (illustrated with red arrows), which include 7 seconds of LED on and 3 seconds of LED off in all cases.

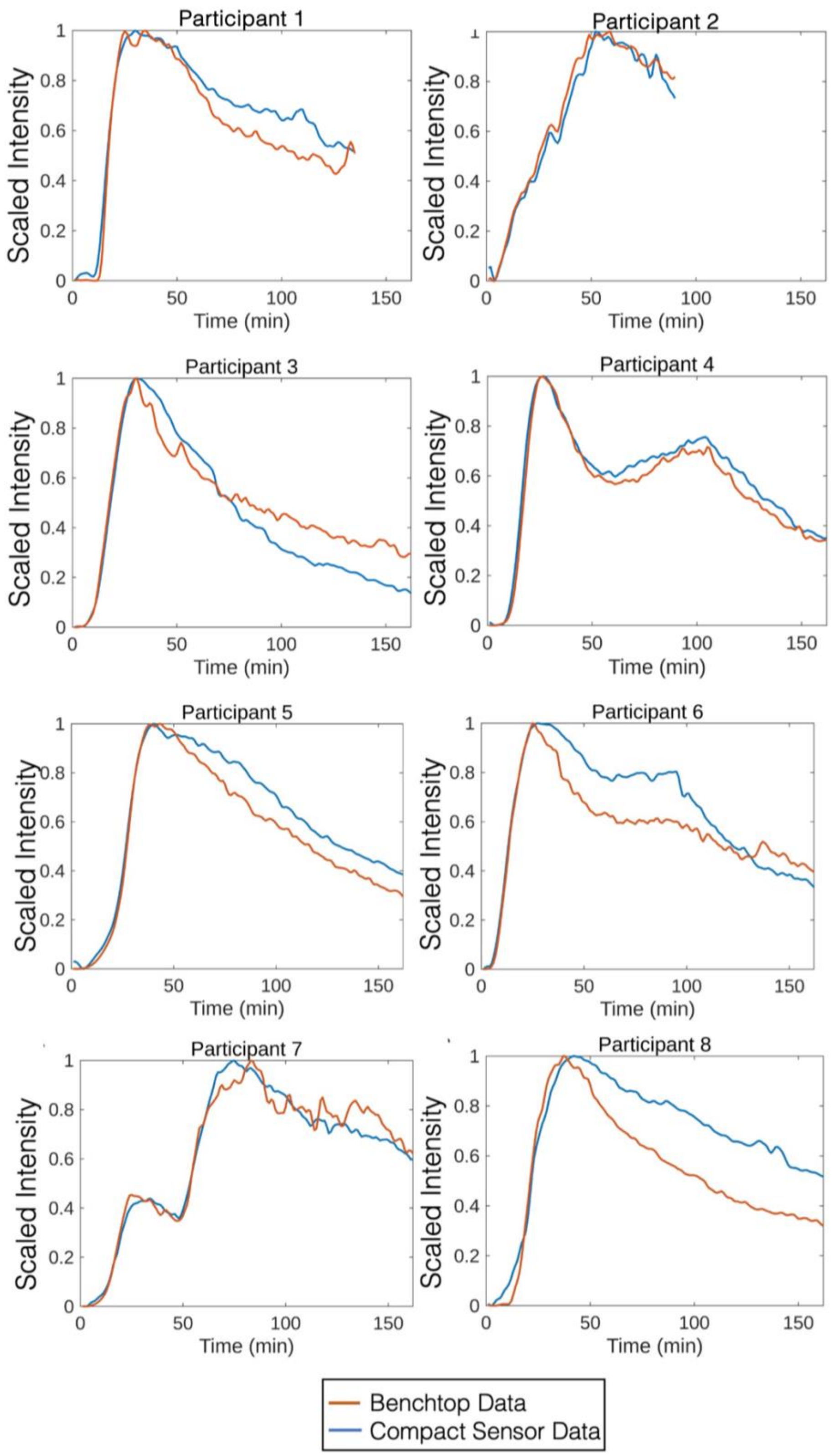

Fig. S5. Individual scaled fluorescence intensity vs. time curves for participants 1-8. Benchtop system – orange; compact sensor – blue. Signals were recorded for a total of 180 minutes, with the exception of participants 1 and 2, where data collection issues necessitated early termination of experiments (after 135 and 90 minutes respectively). The presented fluorescence vs. time curves are scaled to their maximum intensity (from 0–1) in order to address the different absolute values produced by the compact sensor and benchtop system (due to differences in the detection electronics used and the corresponding outputs). This facilitated investigation of correlation between datasets collected with the two devices.

Table S1. Pearson's correlation coefficients and *p*-values for the datasets presented in Fig. 4 and Fig. S5.

Participant	Correlation coefficient (*r*)	Correlation coefficient *p-value*	95% confidence interval (lower and upper bounds)
P1	0.978	<0.001	[0.970 0.984]
P2	0.994	<0.001	[0.992 0.996]
P3	0.964	<0.001	[0.951 0.973]
P4	0.993	<0.001	[0.991 0.995]
P5	0.988	<0.001	[0.984 0.991]
P6	0.935	<0.001	[0.914 0.951]
P7	0.982	<0.001	[0.977 0.987]
P8	0.919	<0.001	[0.892 0.939]
**Mean**	**0.9898**	**<0.01**	**[0.986 0.992]**

Table S2. Parameters extracted from the datasets presented in Fig. 4 and Fig. S5. AUC: area under curve. Peak AUC represents the AUC value calculated from time = 0 minutes until the time at which maximum intensity is observed (Peak Time). To compare the extracted parameters, signals from each participant and system were first scaled to their maximum intensity (i.e. range = 0–1). Parameters for participants 3-8 were extracted from the full 180 minutes of data. Parameters for participants 1 and 2 were extracted from 135 and 90 minutes of data respectively (due to the early termination of data collection explained above). The Table includes Peak Time, Total AUC and Peak AUC values for each participant, as well as the mean and standard deviation (STD) calculated across all participants. The same parameters were also extracted from the mean curves (displayed in Fig. 5B) and are shown at the end of the table (including values from error bars, which correspond to the upper and lower bounds of the shaded regions in Fig. 5B). Note that no error bar values are included for Peak Time as time points for data collection were the same across all participants.

Participants	System	Peak Time (min)	Total AUC	Peak AUC
P1	Benchtop system	35	81.280	17.072
	Compact sensor	30	90.273	13.001
P2	Benchtop system	59	60.832	33.122
	Compact sensor	52	58.721	24.517
P3	Benchtop system	30	78.729	12.089
	Compact sensor	31	70.614	12.674
P4	Benchtop system	26	95.704	8.832
	Compact sensor	27	101.553	10.783
P5	Benchtop system	43	93.097	16.632
	Compact sensor	40	103.599	14.293
P6	Benchtop system	25	99.953	10.977
	Compact sensor	28	109.825	14.284
P7	Benchtop system	83	111.608	39.572
	Compact sensor	75	109.857	32.918
P8	Benchtop system	37	90.674	15.124
	Compact sensor	42	117.328	19.816
**Mean**	**Benchtop system**	**38.968**	**87.727**	**16.882**
	**Compact sensor**	**38.275**	**92.956**	**16.613**
STD	Benchtop system	19.761	15.372	11.098
	Compact sensor	16.317	20.663	7.5684
**Values from mean curves (Fig. 5B)**	**Benchtop system**	**37**	**91.114**	**15.807**
	**Compact sensor**	**39**	**97.749**	**17.755**
Error bars from mean curves (Fig. 5B)	Benchtop system	-	24.594	3.055
	Compact sensor	-	26.559	2.818

Table S3. Statistical analysis comparing benchtop system and compact sensor data using the parameters shown in Table S2. Individual parameter values (from P1 to P8) were used to compare the compact sensor and benchtop system, for Peak Time, Total AUC and Peak AUC respectively. No statistically significant differences are observed, suggesting agreement/correlation between the data collected with the two devices.

Parameter	Test	*P-value*
Peak Time	Student T-test (paired)	0.371
Total AUC	Student T-test (paired)	0.142
Peak AUC	Student T-test (paired)	0.441

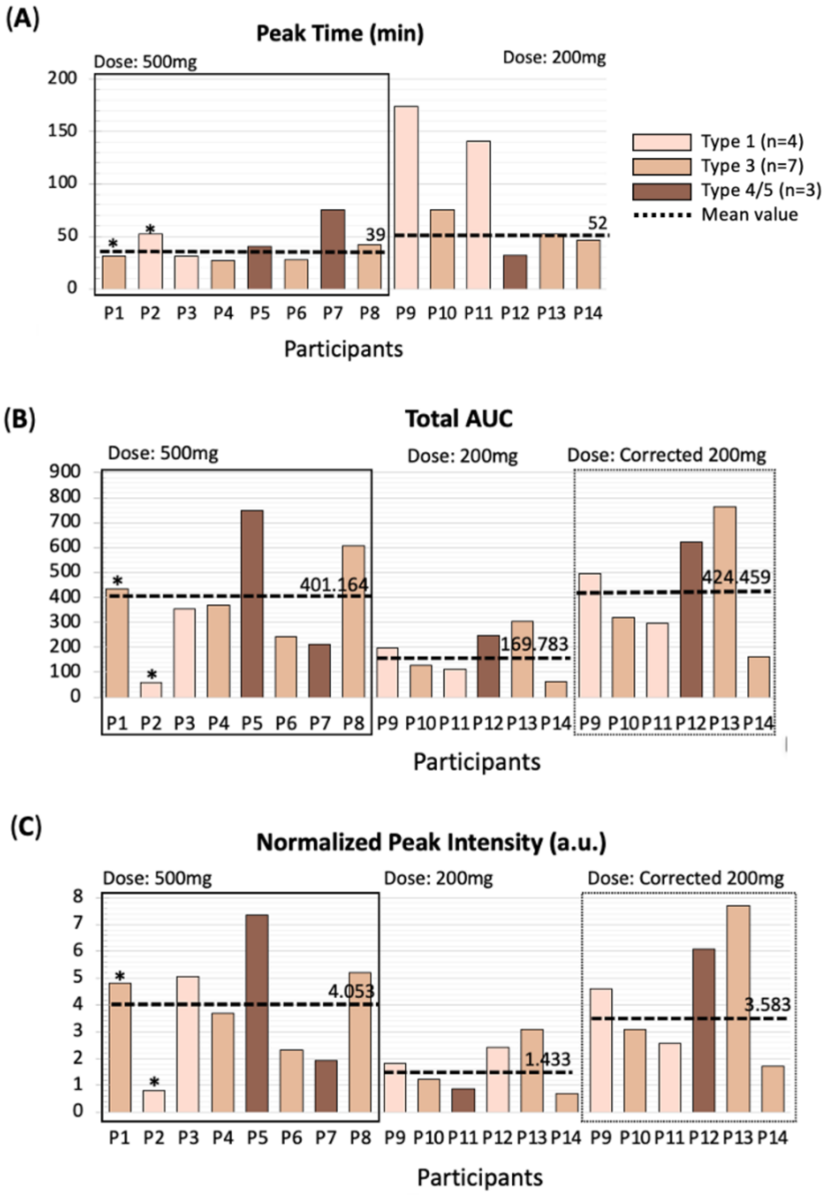

Fig. S6. Peak Time, Total AUC and Normalized Peak Intensity values calculated from the compact sensor datasets for all participants (P1–14). Values for participants 3–14 were extracted from the full 180 minutes of collected data. Due to early termination of data collection (explained above), parameters for participants 1 and 2 (indicated with an *) were extracted from 135 and 90 minutes of data respectively. On each graph, data is classified according to contrast agent dose (including the "Corrected 200 mg" data, where data collected at 200 mg was multiplied by a correction factor of 2.5 to allow comparison to data collected at 500 mg). Bars are color-coded based on the Fitzpatrick skin tone type for each participant (see legend). The numbers of participants in each Fitzpatrick group are shown in the legend. Dotted lines represent the values from the mean fluorescence vs. time curves (presented in Fig. 5B) for each dose condition. (**A**) Peak Time parameter representing the time (in minutes) at which the signal reaches maximum intensity. (**B**) Total AUC. (**C**) Normalized Peak Intensity representing the maximum fluorescence intensity value for each dataset.

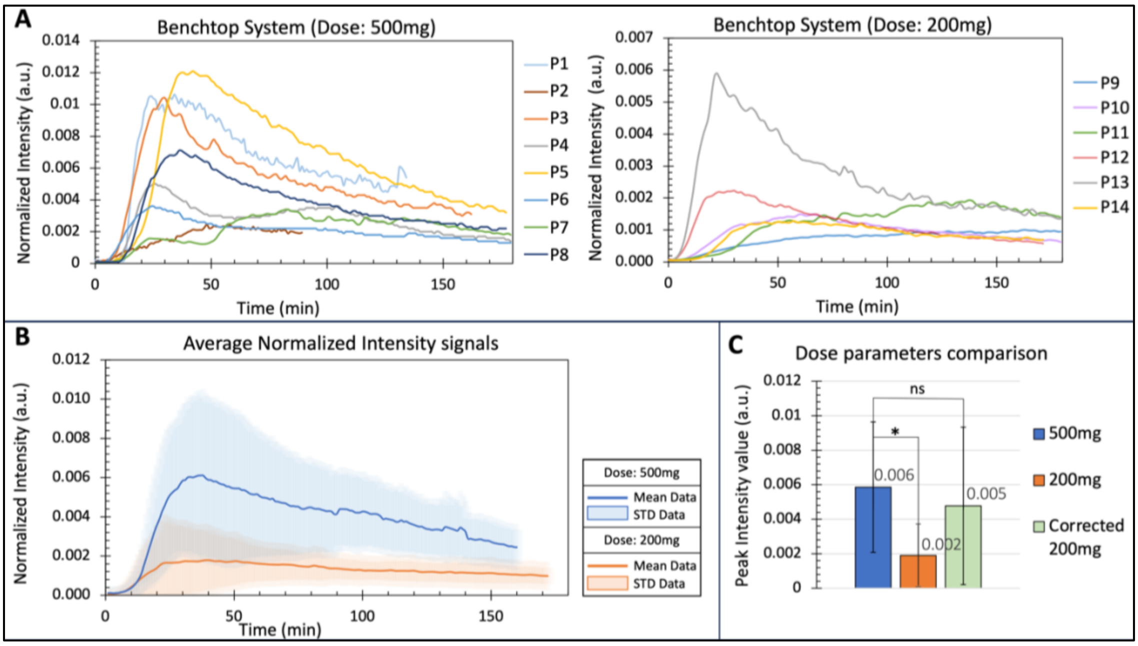

Fig S7. Characterization of benchtop system under different fluorescein dose conditions. (**A**) Time-resolved normalized fluorescence signals for participants 1-8 (left; 500 mg fluorescein in 100 ml water) and participants 9-14 (right; 200 mg fluorescein in 100 ml water). Participant 1 and 2 present shorter signals (135 and 90 minutes respectively) due to data collection issues that necessitated early termination of experiments. (**B**) Mean (± STD) normalized fluorescence vs. time curves for both dose conditions: 500 mg (blue), 200 mg (orange). The irregularities/discontinuities observed at 90 and 135 minutes on the mean normalized fluorescence signal for the 500 mg dose are a result of the shorter data collection times for participants 1 and 2. (**C**) Peak (maximum) intensity values extracted from the mean normalized intensity curves shown in panel B. A statistically significant difference was observed between the 500 mg and 200 mg peak intensity values ($p = 0.0197$). When a correction factor (of 2.5) was applied to the 200 mg peak intensity (“Corrected 200 mg”; green bar), no statistically significant difference was observed when compared against the 500 mg dose ($p = 0.6432$), indicating an approximately linear relationship between dose and peak intensity. * – $p < 0.05$; ns – not significant.

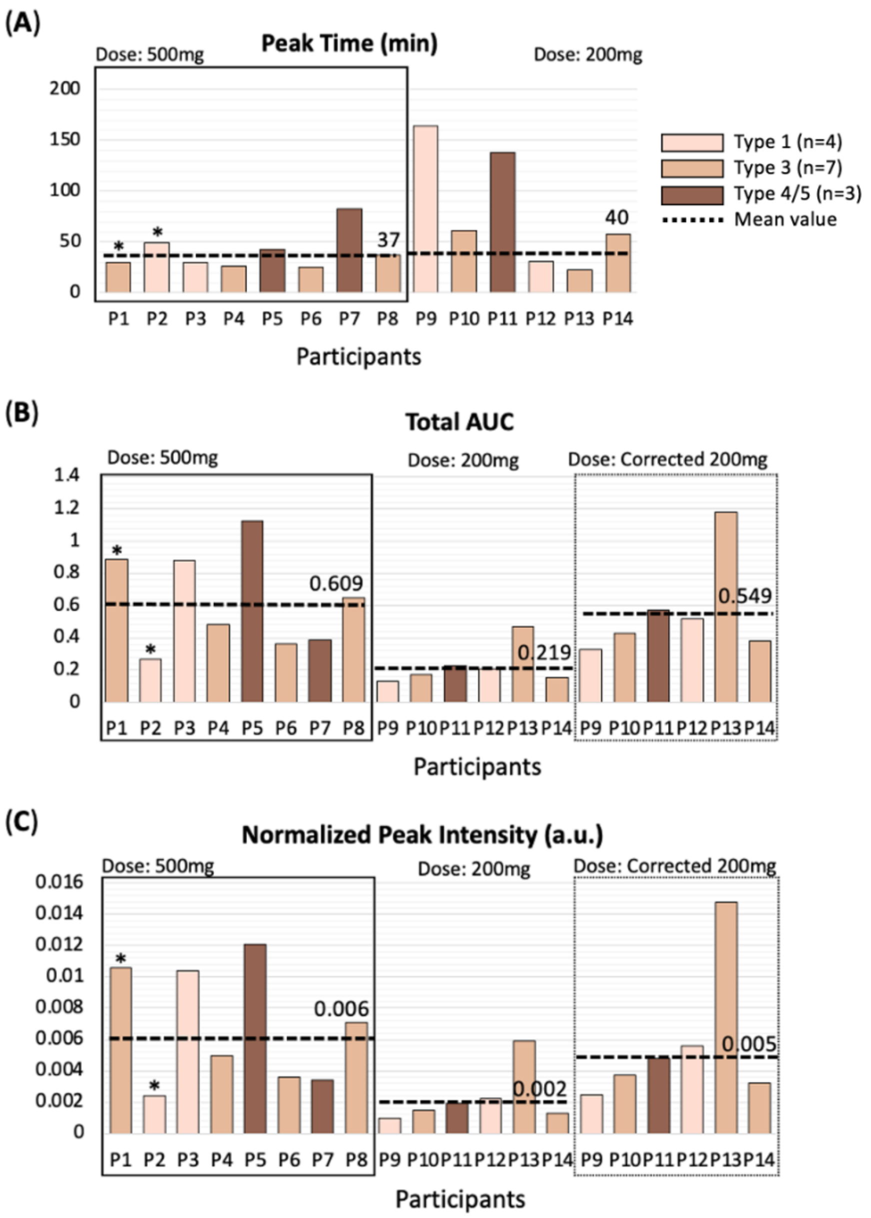

Fig. S8. Peak Time, Total AUC and Normalized Peak Intensity values calculated from the benchtop system datasets for all participants (P1–14). Values for participants 3–14 were extracted from the full 180 minutes of collected data. Due to early termination of data collection (explained above), parameters for participants 1 and 2 (indicated with an *) were extracted from 135 and 90 minutes of data respectively. On each graph, data is classified according to contrast agent dose (including the "Corrected 200 mg" data, where data collected at 200 mg was multiplied by a correction factor of 2.5 to allow comparison to data collected at 500 mg). Bars are color-coded based on the Fitzpatrick skin tone type for each participant (see legend). The numbers of participants in each Fitzpatrick group are shown in the legend. Dotted lines represent the values from the mean fluorescence vs. time curves (presented in Fig. S7B) for each dose condition. (**A**) Peak Time parameter representing the time (in minutes) at which the signal reaches maximum intensity. (**B**) Total AUC. (**C**) Normalized Peak Intensity representing the maximum fluorescence intensity value for each dataset.

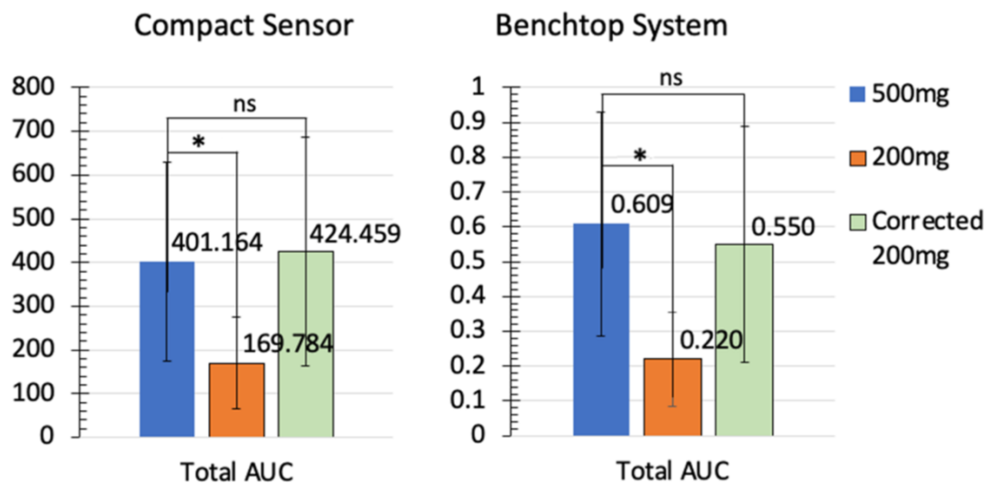

Fig S9. Total AUC values extracted from the mean normalized fluorescence intensity vs. time curves for both devices (left – compact sensor; right – benchtop system). Values were extracted from data shown in Fig. 5B (for compact sensor) and Fig. S3B (for Benchtop system). Statistically significant differences were observed between the 500 mg and 200 mg Total AUC values for both devices (identified by *). When a correction factor (of 2.5) was applied to the 200 mg Total AUC values ("Corrected 200 mg"; green bar), no statistically significant differences were observed when compared against the 500 mg data (for both devices), indicating an approximately linear relationship between dose and Total AUC. * – $p < 0.05$; ns – not significant.

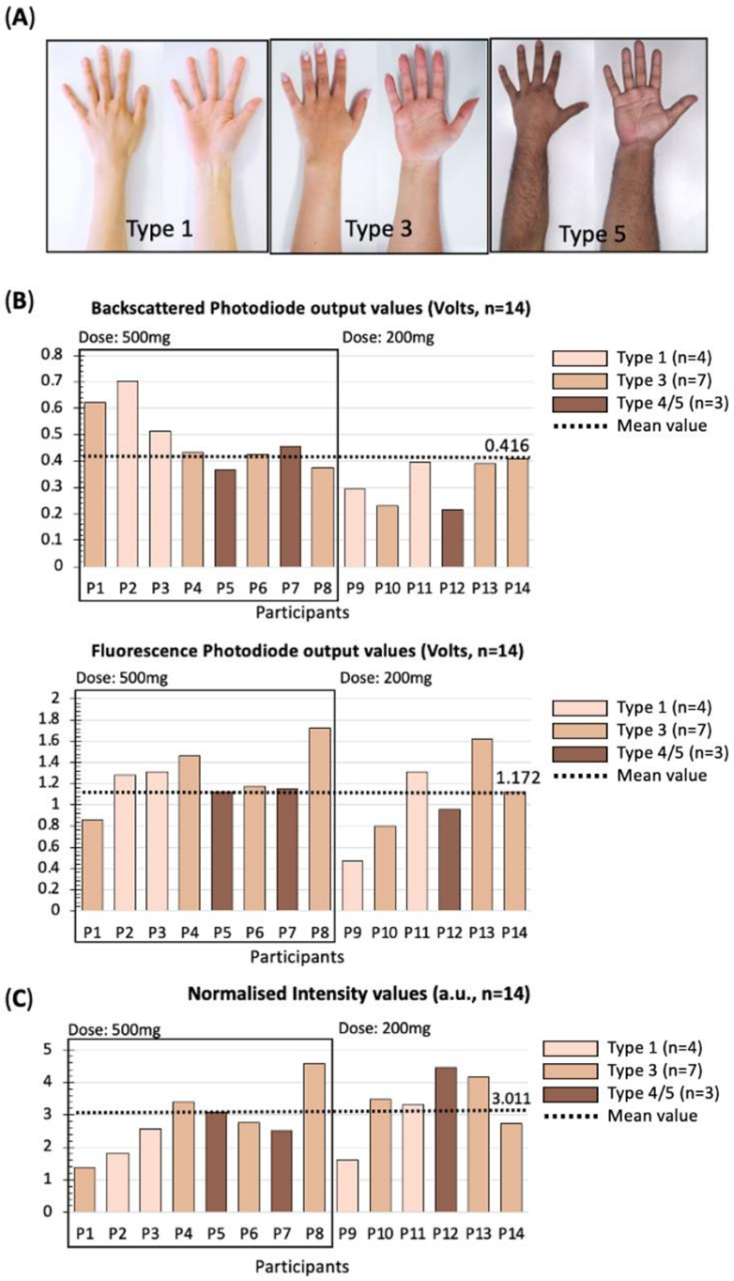

Fig S10. Characterization of compact sensor under different skin tone conditions – individual values. (**A**) Photographs of the hands and arms of three participants representing Fitzpatrick types 1, 3 and 5 (left to right respectively). **(B)** Individual backscattered (upper graph) and fluorescence (lower graph) photodiode values averaged over the first 10 minutes of data (i.e., where no fluorescein signal is present). (**C**) Individual normalized intensity values averaged over the first 10 minutes of data (i.e., where no fluorescein signal is present). Bars are color-coded according to Fitzpatrick skin tone type (see legends). The numbers of participants included in each group are shown in the legends (total number of participants: n=14). Fluorescein doses used for each participant are indicated on the graphs. Dotted lines represent the mean values extracted from the curves averaged across all participants (i.e., with Fitzpatrick classification not considered).
